# Non-Operative Management of Iatrogenic Intraperitoneal Bladder Injury Following a Cesarean Section

**DOI:** 10.7759/cureus.13150

**Published:** 2021-02-05

**Authors:** Saeed N Albukhari, Abdullah Khawaji, Raed A Azhar

**Affiliations:** 1 Medicine, College of Medicine, King Saud Bin Abdulaziz University for Health Sciences, Jeddah, SAU; 2 Urology, International Medical Centre, Jeddah, SAU; 3 Urology, International Medical Centre, King Abdulaziz University, Jeddah, SAU

**Keywords:** iatrogenic bladder injury, cesarean section, conservative treatment, non surgical treatment

## Abstract

Iatrogenic bladder injuries with intraperitoneal extravasations are standardly managed surgically. However, we are presenting a case of iatrogenic intraperitoneal bladder injury developing after a cesarean section that was managed successfully by conservative therapy after tapping and pigtail drainage of 14 days. On the next follow up, which was 14 days later, there was a complete resolution with no signs of injury. Therefore, the trial of conservative approach may prove beneficial to minimize the chances of any invasive interventions in such cases.

## Introduction

Cesarean section (CS) route of delivery has been markedly increasing in Saudi Arabia with an 80.2% increase over a 10-year period reported by the ministry of health (MOH) from 1997 to 2006 [1]. Although it has not been associated with significant risk or injuries for the mother, yet the most injured urological organ during obstetrical and gynecological operations is the bladder [2]. Intraperitoneal, extraperitoneal, and combined injuries are the standard classification of bladder rupture [3]. Furthermore, bladder trauma is considered the commonest cause of bladder rupture by blunt, penetrating, or iatrogenic modality [4]. Moreover, the standard management of intraperitoneal bladder injury (IPBI) is usually surgical repair, while extraperitoneal bladder injury (EPBI) is usually managed conservatively for the majority of those cases [3]. However, the tendency to manage urological trauma conservatively has been increasing to minimize the invasiveness of the procedures on the patient [2]. Herein, we present a case of a female who developed an intraperitoneal bladder injury following a CS and was successfully managed conservatively.

## Case presentation

**Figure 1:**
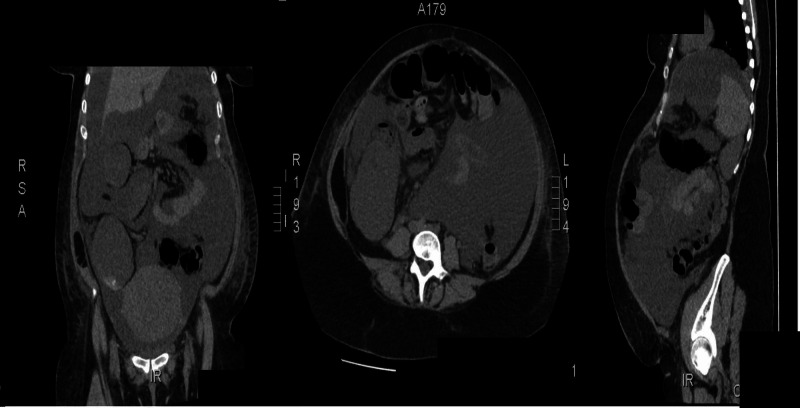
Non-contrast CT scan with large amount of intraperitoneal fluid accumulations

**Figure 2:**
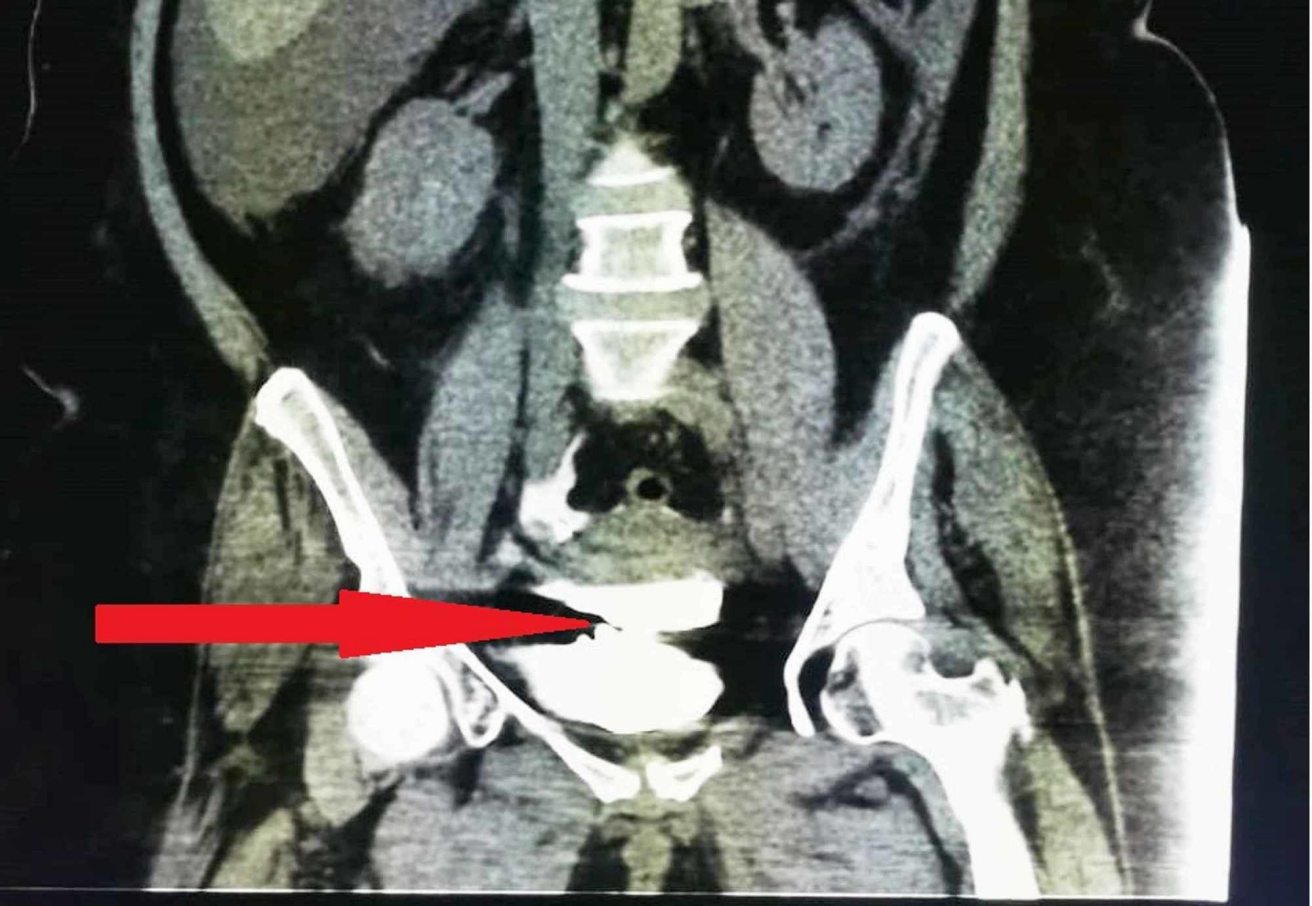
CT cystogram showing a defect in the dome of the urinary bladder with extravasation of contrast to intraperitoneal space

A 35-year-old female, not known to have any chronic illnesses, presented to the urology department after the patient developed sudden hematuria six hours after a lower abdominal cesarean section. Her medical history was insignificant with one previous child who was delivered by CS and no allergies or medications confirmed. Meanwhile, social history was negative for tobacco, alcohol, or any illicit drug use. Upon examination, the patient was afebrile, vitally and hemodynamically stable, while systemic review was unremarkable. Moreover, local examination showed a full flank with mild abdominal distention and mild generalized tenderness without any signs of peritonitis. Regarding her investigations, complete blood count was within normal limits while Renal Function Test (RFT) showed elevated creatinine (3.42 mg/dl) (Table 1). A non-contrasted Computed Tomography (CT) was ordered and a finding of a large amount of intraperitoneal fluid accumulations was reported (Figure 1). After normalization of serum creatinine and glomerular filtration rate (GFR), which was achieved by a foley catheter drainage, a CT cystogram showed a defect in the dome of the urinary bladder with extravasation of contrast to intraperitoneal space (Figure 2).

**Table 1:** Laboratory workup upon first presentation CBC: complete blood count, GFR: glomerular filtration rate, Hb: hemoglobin, WBC: white blood cells, RFT: Renal Function Test

First Day of Presentation		Second Day after Tapping	
CBC		CBC	
WBC	8.5 x 10^9/L	WBC	4.09 x 10^9/L
Hb	10.7 g/dL	Hb	9 g/dL
RFT		RFT	
Serum Creatinine	3.42 mg/dl	Serum Creatinine	0.64 mg/dl
GFR	15	GFR	105

The choice of taking a non-invasive approach was determined after the patient's refusal for any further surgical interventions to be done. Afterwards, tapping off the intraperitoneal fluid was done with an abdominal fluid drain insertion which was inserted under local anesthesia and under the guidance of ultrasound and complete aseptic conditions rather than the standard surgical approach for the removal of the intraperitoneal fluid collection. Moreover, a 6.5 French pigtail catheter was inserted for 14 days to allow for maximum fluid drainage with a plan for surgery in case there is further deterioration. Fluid chemistry showed high creatinine levels (377 mmol/L), indicating the presence of urine in the abdominal area within less than 24 hours. The following day, her condition improved substantially, pain subsided with no symptom or sign of peritonitis, while GFR levels went back to normal (≥ 90 mL/min/1.73 m2). As a result of such improvement, a decision was made to manage the case conservatively by providing maximum fluid drainage with a course of antibiotic (ceftriaxone). On her third day, hematuria level regressed markedly and was considered safe for discharge with a scheduled follow up in two weeks.

**Figure 3:**
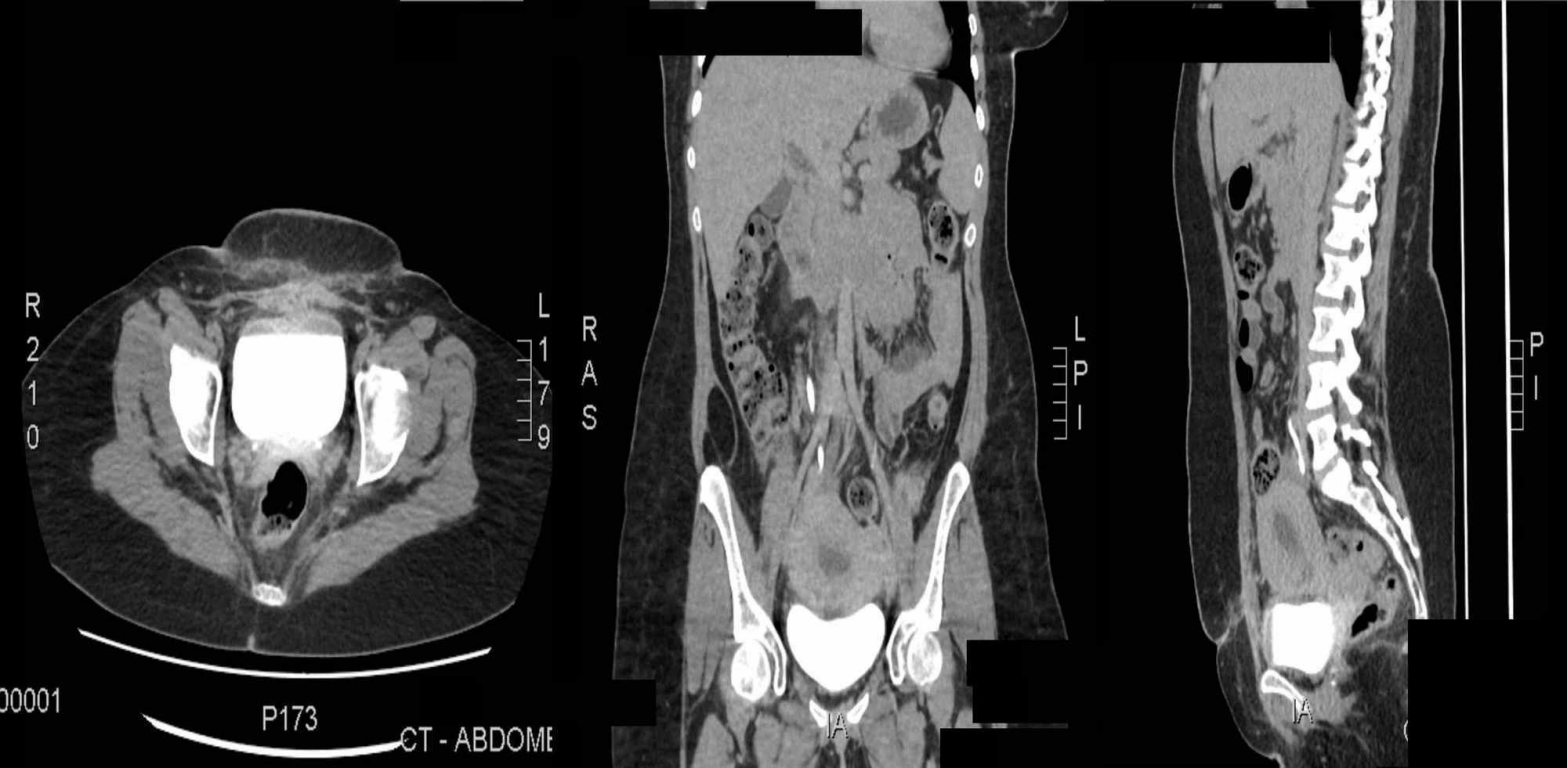
Interval resolution of the abdominopelvic ascites status post drainage

On her follow up, which was done after two weeks, a CT urogram was done and showed an interval resolution of the abdominopelvic ascites and normal opacification of the collecting system with no evidence of contrast extravasation, suggesting no ureteric or urinary bladder injury, and no further medical intervention was needed (Figure 3).

## Discussion

Even though CS surgeries have a low tendency for developing morbidities and mortalities to the mother, they has been associated with urological injuries, with the bladder being the most frequently injured urological organ with incident ranging from 0.08 to 0.94% [2]. Moreover, according to a case-control study done in 2010 for 56,799 women who underwent CS, CS puts the women into greater risk for developing bladder injuries during the surgery [5]. However, trauma (blunt, penetrating, or iatrogenic), spontaneous, and intoxication are the three causes of bladder injuries, with trauma being the most common cause (96%). Meanwhile, bladder ruptures are managed depending on their type, either being intraperitoneal with a 25% occurrence rate, or extraperitoneal with an occurrence of 60-65%) [4]. Aghaways et al. previously reported a similar case of bladder injury that was managed with a Foley catheter and percutaneous intraperitoneal pigtail catheter and omitted the surgical intervention due to the instability of the patient [6]. Afterwards, the patient responded well to conservative management without the need for surgical intervention [6]. Furthermore, it has been documented in the literature that cases of uncomplicated EPBI due to pelvic fractures can be managed conservatively with a large-bore catheter for a three-week duration [7]. This warrants that management of such uncomplicated cases can be achieved without the necessity to surgically intervene. In addition, EPBI management is deemed safe by catheterization alone, while IPBI may necessitate surgical repair [3]. Taking into consideration that the 350 ml contrasted cystography is the diagnostic study for bladder injuries [3], we could not inject a contrast into our patient due to high levels of creatinine on RFT, which was an absolute contraindication and would risk kidney injury. Our patient was anxious, concerned, and looked stable with no signs of sepsis or peritonitis. Since then, our main focus was complete drainage to avoid the surgical approach to protect the patient from potential surgical and anesthetic complications. Moreover, the marked decrease in the creatinine one day after tapping indicates that adequate efficient drainage is enough, so surgery was excluded since the patient had been controlled and managed sufficiently.

Lastly, the choice of choosing to conservatively manage the patient depends on the type of bladder injury, the cystogram and void study, the size of the injury, having complete drainage, and how is the patient's tolerability to such management. EPBI can resolve conservatively with Foley catheter and intra-abdominal catheterization if the injury size is not large to cause major spills and the patient's hemodynamic stability is not deteriorating further during the management. Careful observation of kidney functions is necessary in order to avoid any complications.

## Conclusions

All in all, we present a case of intraperitoneal bladder injury following a CS. Although iatrogenic bladder injuries in CS are usually managed intraoperatively, we decided to manage the patient conservatively respecting the patient autonomy and to avoid surgical and anesthetic complications. Consideration should be given for the conservative approach in the future for any patient with an iatrogenic intraperitoneal bladder injury as long as adequate drainage is performed, and they do not show signs of sepsis, deterioration, or peritonitis.
